# Confirmed malaria cases among children under five with fever and history of fever in rural western Tanzania

**DOI:** 10.1186/1756-0500-4-359

**Published:** 2011-09-13

**Authors:** Humphrey D Mazigo, Wilfred Meza, Emanuella E Ambrose, Benson R Kidenya, Eliningaya J Kweka

**Affiliations:** 1 Weill-Bugando University College of Health Sciences, P.O. Box 1464, Mwanza, Tanzania; 2 Tropical Pesticides Research Institute, Division of Livestock and Human Diseases, Vectors Control, Mosquito section, P.O. Box 3024 Arusha, Tanzania

**Keywords:** Fever, history of fever, parasitological diagnosis, western Tanzania

## Abstract

**Background:**

The World Health Organization recommends that malaria treatment should begin with parasitological diagnosis. This will help to control misuse of anti-malarial drugs in areas with low transmission. The present study was conducted to assess the prevalence of parasitologically confirmed malaria among children under five years of age presenting with fever or history of fever in rural western Tanzania. A finger prick blood sample was obtained from each child, and thin and thick blood smears were prepared, stained with 10% Giemsa and examined under the light microscope. A structured questionnaire was used to collect each patient's demographic information, reasons for coming to the health center; and a physical examination was carried out on all patients. Fever was defined as axillary temperature ≥ 37.5°C.

**Findings:**

A total of 300 children with fever or a history of fever (1 or 2 weeks) were recruited, in which 54.3% (163/300, 95%CI, 48.7-59.9) were boys. A total of 76 (76/300, 25.3%, 95%CI, 22.8 - 27.8) of the children had fever. Based on a parasitological diagnosis of malaria, only 12% (36/300, 95%CI, 8.3-15.7) of the children had *P. falciparum *infection. Of the children with *P. falciparum *infection, 52.7% (19/36, 95%CI, 47.1-58.3) had fever and the remaining had no fever. The geometrical mean of the parasites was 708.62 (95%CI, 477.96-1050.62) parasites/μl and 25% (9/36, 95%CI, 10.9 -- 39.1) of the children with positive *P. falciparum *had ≥ 1001 parasites/μl. On Univariate (OR = 2.13, 95%CI, 1.02-4.43, P = 0.044) and multivariate (OR = 2.15, 95%CI, 1.03-4.49) analysis, only children above one year of age were associated with malaria infections.

**Conclusion:**

Only a small proportion of the children under the age of five with fever had malaria, and with a proportion of children having non-malaria fever. Improvement of malaria diagnostic and other causes of febrile illness may provide effective measure in management of febrile illness in malaria endemic areas.

## Background

In Tanzania, malaria is a leading cause of health service attendance and the disease contributes to approximately 40% of all morbidities reported in children under five presenting in outpatients [1]. One of the control measures against malaria and its related morbidities in Tanzania is by case management through early diagnosis and prompt treatment using effective drugs [1,2]. Microscopy which detects malaria parasites in Giemsa stained thick and thin blood slides [3,4] and Malaria Rapid Diagnostic Tests (mRDTs) which detect malaria parasite antigens in blood samples [2,5-7], are used for malaria diagnosis. In areas where there are no facilities, such as electricity, to support the use of microscopy, RDTs are recommended [5-7]; however, the costs and practicability of introducing these diagnostic facilities in rural areas is a challenge [2]. In areas where these diagnostic facilities are not available, presumptive treatment of all fevers in children under five has been widely practised in managing fevers in Tanzania [8]. The policy of presumptive treatment of malaria for all febrile illnesses has been widely advocated in sub-Saharan Africa, especially in young children [8,9]. Despite this approach reporting beneficial effects among African children [9,10], it has resulted in a large degree of unnecessary use of antimalarials, especially in areas with low transmission [11]. At a time when malaria transmission intensity is reported to be in decline [12-16] and malaria endemic countries are reporting low transmission intensity [15], presumptive treatment may no longer be justifiable. The continuity of presumptive treatment may result in malaria parasites developing resistance against artemesinin based combination therapy (ACT) which are currently used as the first line treatment against malaria in Tanzania [17].

At present, episodes of malaria related fever among children under five are reported to be in decline in Africa [18], and based on this observation, the World Health Organization recommends that malaria treatment should begin with parasitological diagnosis [19-23]. Light microscopy or RDTs offer an inexpensive and practical means of improving malaria diagnosis and treatment in areas of low transmission [2-5]. In addition, parasitological diagnosis and treatment of febrile children based on laboratory confirmed results is cost-effective [24]. However, there is still no consensus on laboratory-confirmed diagnosis versus presumptive treatment for malaria in endemic areas [9,22,25].

There is a strong need for clinicians to base their treatment decisions on laboratory confirmed cases of malaria. Parasitological diagnosis will enable clinicians to report more accurately whether patients presenting with fever or history of fever actually are parasitaemic [22]. Evidence from malaria endemic areas shows that there has been a large decrease in cases of malaria reported; and this, in part, is due to the reliance on laboratory confirmed cases of malaria [2,4,21]. In addition, reliance on laboratory results will reduce the unnecessary use of antimalarials in non-malaria cases, and improve treatment for other potentially fatal causes of fevers. For these reasons, the present study was conducted to assess the prevalence of parasitologically confirmed malaria among children under five, presenting with a fever or a recent history of fever at the Nyasa Health Centre in western Tanzania.

## Methods

### Study area, design and population

The cross-sectional survey was conducted amongst children attending the outpatient clinic at the Nyasa Health Center located in Nzega District, Tabora region, in western Tanzania. The study was conducted between August and October 2010. Nzega district is located in a holoendemic area and intense malaria transmission is observed during the long rain season (February-May).

The inclusion criteria for the study subjects were: (1) children who sought treatment at the health center and had fever/history of fever in the past 1-2 weeks, (2) Parents/guardians consent for their children to participate in the study, (3) Children aged between 2 months and 60 months, and (4) no history of treatment with anti-malarial drugs in the past two weeks.

### Data collection

Parents/guardians of eligible children gave written informed consent to allow their children to participate in the study. Children under five were consequently recruited into the study until the sample size of 300 was reached. A qualified clinician used a pre-tested Swahili translated questionnaire to collect patient's demographic information and the reasons why he/she was brought to the health center. The clinician conducted a physical examination on all patients and checked for pallor, spleen enlargement, jaundice and other abnormalities. The axillary body temperature was measured using a digital clinical thermometer. Fever was defined as body temperature ≥ 37.5°C.

A finger prick blood sample was obtained and one thin and two thick blood smears were prepared and stained with 10% Giemsa. The diagnosis and management of malaria was based on initial readings of one of the thick blood smears at the health centre. Management of malaria was done in accordance with the Tanzania Malaria Treatment Guidelines. The final reading of the Giemsa stained slides was done at Weill-Bugando University College of Health Sciences which was based on a rigorous quality control system. A second microscopist re-examined all the stained blood smears and any disagreement between the first and second microscopist was resolved by a third microscopist. In addition, the third microscopist examined 10% of positive and negative slides which were selected randomly. The microscopist identified malaria parasite stages and speciated the malaria parasites. The Giemsa stained smears were examined at 100× objectives under oil immersion and a slide with the presence of trophozoites or any other asexual form of *Plasmodium *species was declared as positive. Slides were declared as negative if no asexual parasites were found after examining 100× objectives. *P. falciparum *parasite density was determined according to the number of parasites per 200 WBC (white blood cells), assuming a total WBC count of 8,000/μL.

### Data analysis

Data were analysed using Stata Version 11 (Stata Corp, College station, Texas, USA). An estimate of relative frequency and means for demographic data and outcome of interest was derived. In order to determine independent predictors of malaria in febrile children, numerous variables were assessed, including demographic characteristics, symptoms and clinical signs. Univariate analysis was employed to identify variables with a *P*-value < 0.2 be included in the multivariate model. Stepwise backward logistic regression was used to determine whether these variables were independent predictors of malaria in children. Odds ratios with 95% Confidence intervals were used to measure the strength of association at statistical significance level of *P *< 0.05.

### Ethical clearance

The study received ethical clearance from the faculty of medicine, Weill-Bugando University College of Health Sciences, Mwanza, Tanzania.

## Results

A total of 300 children under five with fever or history of fever participated in the study. The median age of the enrolled children was 13 months (range-minimum-maximum: 8 - 23.5 months). Of the 300 children under five, 54.3% (163/300, 95%CI, 48.7-59.9) were boys and 45.7% (137/300, 95%CI, 40.1-51.3) were girls. Parents/guardians reported fever or history of fever as part of the illness in all the children, although during physical examination only 25.3% (76/300, 95%CI, 22.8-27.8) had an axillary temperature ≥ 37.5°C.

Parasitological diagnosis of malaria parasites revealed that out of the 300 children under five, only 12% (36/300, 95%CI, 8.3-15.7) had positive slide readings. Of these, 52.7% (19/36, 95%CI, 47.1-58.3) had fever and 42.7% (17/36, 95%CI, 26.7-58.7) had no fever on physical examination. The distribution of positive malaria slide readings between girls and boys was not statistically significant (χ^2 ^(1) = 2.7248 *P *= 0.099). Table 1, shows the distribution of demographic characteristics, symptoms and clinical signs of children under five with and without malaria.

**Table 1:** Distribution of demographic characteristics, symptoms and clinical signs of children under five with and without malaria in western Tanzania.

Variable	Malaria positive		Malaria negative	
	N = 36	(%)	N = 264	(%)
**Sex**				

Female	21	(58.3)	116	(43.9)

Male	15	(41.7)	148	(56.1)

**Age (in years)**				

Below 1 year	12	(33.3)	136	(51.5)

Above one year	24	(66.7)	128	(48.5)

**Symptoms and clinical signs**				

Fever and other symptoms/clinical signs	35	(97.2)	247	(93.6)

Other symptoms and clinical signs without fever	1	(2.8)	17	(6.4)

**Key: **Fever only = define as body temperature ≥ 37.5°C

Other symptoms and clinical signs included:- vomiting, diarrhoea, dysentery, dehydration, lower limb swelling, difficulty in breathing, wounds and abdominal discomfort.

All the *Plasmodium *positive children were positive for *P. falciparum *and the geometrical mean of parasite density was 708.6 (95%CI, 477.9 -- 1050.6) parasites/μl. Among the *P. falciparum*-positive children, 52.8% (19/36, 95%CI, 36.8-68.8) had 1-500 parasites/μl, 22.2% (8/36, 95%CI, 8.7-35.7) had between 501 -- 1000 parasites/μl and 25% (9/36, 95%CI, 10.9-39.1) had ≥ 1001 parasites/μl. There was no statistical significance on the distribution of positivity and parasite density by age (χ^2 ^= 0.5684, *P *= 0.753) and sex (χ^2 ^= 1.4079, *P *= 0.495). Table 2 shows the distribution of positivity and parasites density according to sex and age.

**Table 2:** The distribution of malaria parasites positivity and parasites density by age and sex among 36 children with positive malaria blood slides

		Malaria parasite density (parasite/μl)			χ^2^	*P*-value
	
Variable		1 - 500	501 - 1000	≥ 1001		
		
Sex	Female (N = 21)	10 (47.6%)	5 (23.8%)	6 (28.6%)	0.578	0.753
			
	Male (N = 15)	9 (60%)	3 (20%)	3 (20%)		
Age	Below 1 year (N = 24)	11 (45.8%)	6 (25%)	7 (29.2%)	1.408	0.495
			
	Above 1 year (N = 12)	8 (66.7%)	2 (16.7%)	2 (16.7%)		

Table 3 shows the results of logistic regression analysis of predictors of malaria among the study participants. On Univariate (OR = 2.13, 95%CI, 1.02-4.43, *P *= 0.044) and multivariate (OR = 2.15, 95%CI, 1.03-4.49, *P *= 0.042) analysis, only children above one year of age were associated with malaria infections.

**Table 3:** Univariate and multivariate analysis of predictors of malaria among 300 children under five with fever or history of fever attending the Nyasa health center, western Tanzania

	Unadjusted (univariate)			Adjusted (multivariate)		
**Variable**	**OR**	**95%CI**	***P*- value**	OR*	**95%CI**	***P*-value**

**Age**						

Age below 1 year	1					

Age above 1 year	2.13	1.02 -- 4.43	0.044	2.15	1.03 -- 4.49	0.042

**Sex**						

Female	1					

Male	0.55	0.27 -- 1.12	0.102	0.55	0.27 -- 1.12	0.102

**Fever (body temperature > 37.5°C)**						

No	1					

Yes	1.15	0.53 -- 2.52	0.719	-	-	-

**Reported fever**						

No	1					

Yes	2.40	0.31-18.67	0.400	-	-	-

**Duration of fever**						

≤ 3 days	1					

> 3 days	0.86	0.39 -- 1.93	0.728	-	-	-

**Paleness**						

No	1					

Yes	1.48	0.53 -- 4.13	0.457	-	-	-

**Jaundice**						

No	1					

Yes	1.05	0.13 -- 8.78	0.965	-	-	-

## Discussion

In this study almost 12% of all the study population had malaria positive slides. In all the children under five with malaria, *P. falciparum *was the most predominant species in the study area as well as many parts of Tanzania and Africa [26,27]. The prevalence of *P. falciparum *malaria observed in our study was higher than 6% reported in Pakistan [28] and lower than 56.9% reported in similar study in Nigeria [27]. In Gabon, about 40% of the children presenting at a hospital with fever or history of fever had a *P. falciparum-*positive blood film [29]. The variation in prevalence of *P. falciparum *malaria among the study population could be attributed in part to the difference in malaria transmission patterns, season of conducting the study and the use of malaria prevention tools [30,31].

In our study, a proportion of children under the age of five with *P. falciparum *infections had no fever on physical examination. Similar findings have been reported from malaria endemic areas [27,28]. The presence of malaria parasites without febrile illness could be explained in part by the acquired protective immunity. In endemic areas, protective immunity against malaria is acquired with an increase in exposure to malaria parasites; which increases with age [32]. Because of this, a proportion of children under the age of five who have acquired partial immunity are likely to be asymptomatic or present with periodic fever [32].

Several factors have been described to predict malaria in children under five living in malaria endemic countries [33,34]. Among the identified predictors are fever, duration of fever, intermittent fever and malaria parasitaemia [33,34]. Other predictors reported are signs and symptoms such as headache, anaemia and vomiting [33,34]. However, there is no consensus on this topic [35]. In our study, age (above one year) was the only predictor of malaria in the study participants. The presence of fever or history of fever did not predict malaria. Similar findings have been reported in the Gambia, where the use of fever or history of fever resulted in over-diagnosis of malaria [33]. This was in contrast to a similar study which reported that fever or history of fever with asexual parasitaemia of any density, had higher sensitivity and specificity for diagnosis of malaria [36]. This is to say that parasitological diagnosis of malaria in patients with fever or history of fever remains important in malaria endemic areas.

In our study, a proportion of children under the age of five years presented with fever but had malaria negative blood slides. This indicated that there could be other causes of fever among the febrile children under five in the present study area. At a time when the proportion of malaria-related fevers are reported to be significantly declining in many parts of Africa [18], thus indicating increasing numbers of fevers related to other causes amongst children under five [18], the shift from presumptive diagnosis to parasitological diagnosis is recommended [19-23]. Conversely, in order for the febrile patients to benefit from a full parasitological investigation, reliable and accessible diagnostic facilities are needed. To maximize malaria management, microscopy or RDTs must be made available and utilized appropriately at all level of health facilities. In addition, behaviour change of health workers from the traditional thinking that every fever is malaria is required; more so at this time when some clinicians have lost trust in laboratory results [37]. Health care providers must use microscopy or RDT results to guide anti-malarial treatment practices in a rational way.

Our study is subject to limitations. The cross sectional nature and the small sample size of the children under the age of five years, especially those with fever and malaria parasites, may have contributed to the lack of temporal association between malaria and other variables. In addition, a relatively small sample of the children under five who participated in the study may not be a complete representation of all the children under five who seek health services at the study health centre. Furthermore, the present study was conducted during the dry season, during which malaria transmission is known to be low. This may have contributed to the low prevalence of *P. falciparum *malaria observed in the present study participants, despite the area being located in holoendemic area [30,31,34]. Further investigation on malaria prevalence, with comparisons between seasons, predictors of malaria and use of malaria intervention tools in the present study area are warranted.

In conclusion, only a small proportion of febrile children under five were parasitologically positive for *P. falciparum *malaria and a proportion of the febrile children had malaria negative blood slides. Age (above one year) was the only association with malaria infections. To avoid the current practice of treating every fever with anti-malarial drugs, it is important to improve malaria diagnosis and the diagnosis of non-malaria fever. We recommend that health policy makers consider increasing microscopic diagnoses or malaria Rapid Diagnostic Tests (RDTs) to rural health facilities.

## Competing interests

The authors declare that they have no competing interests.

## Authors' contributions

HDM, EJK and EEM designed the study. WM carried out the clinical examination of the children and interviewed the parents. BRK and HDM conducted the data analysis and drafted the manuscript. All authors read and approved the last version of the manuscript for submission.
